# The CBP/β-Catenin Antagonist, ICG-001, Inhibits Tumor Metastasis via Blocking of the miR-134/ITGB1 Axis-Mediated Cell Adhesion in Nasopharyngeal Carcinoma

**DOI:** 10.3390/cancers14133125

**Published:** 2022-06-25

**Authors:** Luo Chen, Yiu Chun Chiang, Lai Sheung Chan, Wai Yin Chau, Maria Li Lung, Michael Kahn, Kwok Wai Lo, Nai Ki Mak, Hong Lok Lung

**Affiliations:** 1 Department of Biology, Faculty of Science, Hong Kong Baptist University, Hong Kong SAR, China; chenluo2cn@outlook.com (L.C.); cycshawn@gmail.com (Y.C.C.); nkmak@hkbu.edu.hk (N.K.M.); 2 Department of Chemistry, Faculty of Science, Hong Kong Baptist University, Hong Kong SAR, China; ivy_chan@hkbu.edu.hk (L.S.C.); wychau@hkbu.edu.hk (W.Y.C.); 3 Department of Clinical Oncology, University of Hong Kong, Pokfulam, Hong Kong SAR, China; mlilung@hku.hk; 4 Department of Molecular Medicine, Beckman Research Institute at City of Hope, Duarte, CA 91010-3000, USA; mkahn@coh.org; 5 Department of Anatomical and Cellular Pathology, The Chinese University of Hong Kong, Hong Kong SAR, China; kwlo@cuhk.edu.hk; 6 State Key Laboratory of Translational Oncology, The Chinese University of Hong Kong, Hong Kong SAR, China; kwlo@cuhk.edu.hk

**Keywords:** ICG-001, miR-134, *ITGB1*, NPC, Wnt

## Abstract

**Simple Summary:**

Metastatic nasopharyngeal carcinoma (NPC) is incurable and remains the main cause of NPC death. Our previous studies found that the CBP/β-catenin Wnt antagonist, IGC-001, could inhibit the primary tumor formation of NPC tumor cells. Here, we further explored the anti-metastatic activity of ICG-001. We started by screening a panel of microRNAs that are related to epithelial–mesenchymal transition and cancer stem cell phenotypes; both properties can contribute to tumor metastasis. MicroRNA-134 was found to be consistently upregulated by ICG-001. The role of miR-134 in NPC is largely unknown but some studies found an association between low expression of miR-134 and poor prognosis. We examined the role of miR-134 in NPC with both in vitro and in vivo models and found that miR-134 could inhibit cancer cell adhesion, migration, and invasion. Our study provided a functional explanation for the poor prognosis observed in NPC patients with low or loss of miR-134 expression in their tumors and showed that modulation of the Wnt signaling by ICG-001 could effectively inhibit NPC metastasis via the miR-134/*ITGB1* axis.

**Abstract:**

Nasopharyngeal carcinoma (NPC) is an Epstein–Barr virus (EBV)-associated malignancy ranking as the 23rd most common cancer globally, while its incidence rate ranked the 9th in southeast Asia. Tumor metastasis is the dominant cause for treatment failure in NPC and metastatic NPC is yet incurable. The Wnt/β-catenin signaling pathway plays an important role in many processes such as cell proliferation, differentiation, epithelial–mesenchymal transition (EMT), and self-renewal of stem cells and cancer stem cells (CSCs). Both the EMT process and CSCs are believed to play a critical role in cancer metastasis. We here investigated whether the specific CBP/β-catenin Wnt antagonist, IGC-001, affects the metastasis of NPC cells. We found that ICG-001 treatment could reduce the adhesion capability of NPC cells to extracellular matrix and to capillary endothelial cells and reduce the tumor cell migration and invasion, events which are closely associated with distant metastasis. Through a screening of EMT and CSC-related microRNAs, it was found that miR-134 was consistently upregulated by ICG-001 treatment in NPC cells. Very few reports have mentioned the functional role of miR-134 in NPC, except that the expression was found to be downregulated in NPC. Transient transfection of miR-134 into NPC cells reduced their cell adhesion, migration, and invasion capability, but did not affect the growth of CSC-enriched tumor spheres. Subsequently, we found that the ICG-001-induced miR-134 expression resulting in downregulation of integrin β1 (*ITGB1*). Such downregulation reduced cell adhesion and migration capability, as demonstrated by siRNA-mediated knockdown of *ITGB1*. Direct targeting of *ITGB1* by miR-134 was confirmed by the 3′-UTR luciferase assay. Lastly, using an in vivo lung metastasis assay, we showed that ICG-001 transient overexpression of miR-134 or stable overexpression of miR-134 could significantly reduce the lung metastasis of NPC cells. Taken together, we present here evidence that modulation of Wnt/β-catenin signaling pathway could inhibit the metastasis of NPC through the miR-134/*ITGB1* axis.

## 1. Introduction

Nasopharyngeal carcinoma (NPC) is a malignancy arising from the epithelium of nasopharynx. In endemic regions, such as southern China, the incidence rate reaches 25 per 100,000 persons per year [1]. As of 2018, NPC was the 23rd most common cancer globally in terms of incidence, while its incidence rate ranked in 9th place in southeast Asia [1]. Primary NPC is treated mainly with radiotherapy and currently, the 5-year survival rate averages around 80% [2,3]. However, 15–58% of NPC patients will experience recurrent disease, where 5-year survival rate drops to 20% [4]. In addition, 20–30% of patients with locally advanced NPC display metastasis within 3 years with a median overall survival duration of 12–15 months under treatment with palliative chemotherapy [5,6]. Therefore, therapeutically targeting the metastasis of NPC could potentially help to improve the overall survival of NPC patients.

The Wnt/β-catenin signaling pathway plays an important role in many processes such as cell proliferation, differentiation, epithelial–mesenchymal transition (EMT), and self-renewal of stem cells and cancer stem cells (CSCs) [7,8]. EMT is a crucial step in cancer metastasis. During EMT, apical-basal polarized epithelial cells, which normally interact with the basal membrane, switch into front-rear polarized mesenchymal cells with enhanced migration and invasion capability [9,10]. Wnt/β-catenin target genes such as *BOP1*, *CKS2*, *NFIL3*, and *L1CAM* have been shown to induce EMT and promote metastasis in colon cancer [11,12]. CSCs are hypothesized to be a small fraction of the bulk cancer cells, which constitute the origin of most human tumors [13]. Studies have found association between expression of CSC markers and higher metastatic capabilities in breast [14,15] and pancreatic [16] cancers. In NPC, the canonical Wnt/β-catenin signaling pathway was found to be hyperactivated [17,18], mainly due to aberrant methylation at the promoter regions of negative Wnt regulators such as *WIF1*, *DKK1/2*, and *SFRP2*/*SFRP5* [18]. Due to the aberrant status of Wnt/β-catenin signaling in NPC and its impact on EMT and CSCs, therapeutic blockade of Wnt/β-catenin signaling may provide a solution to control NPC metastasis.

Activation of canonical Wnt/β-catenin signaling leads to nuclear accumulation of β-catenin, which activates the transcription of downstream genes to direct cell proliferation and determine cell fate. β-catenin could either interact with the coactivator CREB-binding protein (CBP) for the initiation of self-renewal/proliferation or interact with a homologous coactivator p300 for the initiation of cell differentiation [19,20]. ICG-001 is a small molecule that specifically blocks the binding between CBP and β-catenin, thereby favoring p300/β-catenin binding and driving cell differentiation [21].

MicroRNAs (miRNAs) are non-coding RNAs with an average length of ~22 nucleotides. They are transcribed by RNA polymerase II/III and processed from primary miRNAs (pri-miRNAs) into precursor miRNAs (pre-miRNAs) by a DGCR8/Drosha complex. The processed pre-miRNAs are then exported into the cytoplasm by an exportin 5 (XPO5)/RanGTP complex, where the RNase III endonuclease Dicer cleaves them into amature miRNA duplex. The strands of the mature miRNA can then be loaded into the Argonaute (AGO) family of proteins to form the minimal miRNA-induced silencing complex (miRISC) [22]. The expression of miRNAs can be regulated by long non-coding RNAs (lncRNAs) and circular RNAs (circRNAs) [23]. Furthermore, lncRNAs and circRNAs can affect the function of miRNAs via sponging miRNAs and inhibit its binding with target mRNAs [23]. In most cases, miRNAs interact with the 3′ UTR of target mRNAs via complementarity of nucleotides to suppress gene expression. Full complementarity between a miRNA and miRNA response elements (MREs) in the target mRNA induces target mRNA cleavage, while partial complementarity induces translational inhibition [22].

Micro RNAs have been reported in numerous studies to regulate EMT and CSC maintenance via targeting various components of the Wnt/β-catenin signaling [24,25]. In contrast, much fewer studies have addressed the modulation of miRNAs by Wnt/β-catenin signaling [26]. In our previous studies, ICG-001-mediated blockage of canonical Wnt/β-catenin signaling could inhibit the growth of CSC-enriched NPC tumor spheres via the miR-145/*SOX2* axis [27] and inhibit the migration of NPC cells via the miR-150/*CD44* axis [28] and the miR-96/*EVI1*/miR-449a axis [29]. Here we further investigated the anti-cancer effects of ICG-001 in NPC with a focus on its role in tumor metastasis.

We found that in addition to suppression of migration, ICG-001 treatment could reduce the cell–cell adhesion capability of NPC cells, which is a key requirement for migrating cancer cells to invade their destination tissue. Through a screening of EMT and CSC-related miRNAs (we have previously reported the NPC CSCs-related miRNAs [30]), it was found that miR-134 was consistently upregulated by ICG-001 treatment in NPC cells. To our knowledge, only two reports have studied miR-134 in NPC. Firstly, it was found that one of its products, miR-134-5p, was negatively regulated by *PCAT7* in NPC, which contributes to worse prognosis [31]. A later study showed that miR-134-5p is downregulated in NPC circulating small extracellular vesicles and such downregulation together with upregulation of miR-205-5p and miR-409-3p could serve as a diagnostic marker for NPC [32]. Despite its close relation with NPC prognosis and diagnosis, the functional role of miR-134 in NPC is not determined. In this study, we examined the functional role of miR-134 in NPC and have found that miR-134 can target integrin β1 (*ITGB1*) in NPC. Through downregulating the expression of *ITGB1*, miR-134 inhibits the adhesion and migration capability of NPC cells, leading to reduced metastatic capability of NPC cells in an in vivo model. Taken together, we showed that ICG-001 could suppress NPC metastasis via the miR-134/*ITGB1* axis.

## 2. Materials and Methods

### 2.1. Cell Culture and Chemicals

The EBV-positive C666-1 and EBV-negative HONE-1, HK-1 NPC cell lines were cultured as previously described [28,33]. Cell lines were authenticated by and obtained from the Hong Kong NPC AoE Cell Line Repository. HPMEC cells were purchased from PromoCell (Heidelberg, Germany). A stock solution of ICG-001 (20 mM) was prepared in DMSO and the structure was described previously [34].

### 2.2. Tumor Cell–Extracellular Matrix Adhesion Assay

The cell–matrix adhesion assay was modified based on previously described methods [35]. The cells were pre-treated with ICG-001 or pre-transfected with miR-134. Afterwards, equal numbers of NPC cells (control and treatment) were seeded onto a 96-well culture plate pre-coated with the extracellular matrix component fibronectin (10 μg/mL) (ThermoFisher Scientific, Waltham, MA, USA). The cells were then incubated at 37 °C for 4 h to allow for cell–matrix attachment, followed by PBS (Gibco, New York, NY, USA) rinsing for 3 times. Adhered cells were fixed with paraformaldehyde for 15 min. Afterwards, 0.5% of crystal violet solution was used to stain the cells by incubation at room temperature for 1 h with gentle shaking. Excessive crystal violet was then washed away by PBS rinsing for 3 times. Afterwards, images of crystal violet-stained cells were captured with a bright field microscope. For quantification, 10% acetic acid was used to dissolve the crystal violet and the intensity of crystal violet was measured using a spectrometer.

### 2.3. Adhesion of NPC to Lung Vascular Endothelial Cells

HONE-1 and primary Human Pulmonary Microvascular Endothelial Cells (HPMEC) were used to study the cell–cell adhesion property of NPC cells after ICG-001 treatment. HONE-1 and HPMEC were initially transfected with GFP-expressing vector and RFP-expressing vector, respectively. HPMEC was cultured in a gelatin (10%)-pre-coated-T25 flask for the cells to attain around 90% confluency. Then, 2 × 10^5^ of HONE-1 cells pre-treated with ICG-001 or corresponding solvent control were added to the T25 culture flask containing HPMEC. Subsequently, the cells were incubated for 4 h at 37 °C to allow for cell–cell attachment. PBS was used to wash away floating cells for three times. Images of HONE-1 cells adhering to HPMEC were taken and fluorescence intensity were then quantified by Image J.

### 2.4. Quantification of RNA Expression by Quantitative Polymerase Chain Reaction (qPCR)

Total RNA was extracted using TRIzol Reagent (Invitrogen, Waltham, MA, USA), according to manufacturer’s instruction. For quantification of *ITGB1* mRNA expression, cDNA was synthesized using the M-MLV reverse transcription kit (Promega #M1701, Madison, WI, USA) following manufacturer’s instruction. The *ITGB1* primer sequences were obtained from a previous study [36] (forward: 5′-GACTGATCAGTTCAGTTTGCTGTGTGTTT-3′; reverse: 5′-CCCTGCTTGTATACATTCTCCACATGATTT-3′). The expression of *ITGB1* was normalized against *β-actin* (forward: 5′-TGGATCAGCAAGCAGGAGTATG-3′; reverse: 5′-GCATTTGCGGTGGACGAT-3′). For quantification of miR-134, TaqMan MicroRNA Reverse Transcription Kit (Applied Biosystems, Waltham, MA, USA) was used in reverse transcription. Real-time PCR (qPCR) was subsequently performed in a StepOnePlus Real-time PCR System using TaqMan2X Universal PCR Master Mix (No AmpErase UNG) (Applied Biosystems). Primers for miR-134 (Assay ID:001186) were obtained from TaqMan MicroRNA assays. For normalization, small nuclear RNA RNU6B(U6) was used as an internal control and Taqman primers for U6 were used (Assay ID:001093) for the detection. The relative expression of miR-134 was calculated by 2^−ΔΔCt^ method.

### 2.5. Transwell Migration Assay

Cell migration assay was performed as described previously [29]. Briefly, NPC cells with/without treatment were seeded onto 6.5 mm transwell inserts with 8.0 μm pores polycarbonate membrane (Corning, #3442, Corning, NY, USA). The cells were allowed to migrate for 24 h. Afterwards, the migrated cells were fixed with 4% paraformaldehyde followed by 0.2% Triton-X treatment for permeabilization. Finally, 4,6-Diamidino-2-phenylindole (DAPI) (Sigma-Aldrich, St. Louis, MO, USA) was used to stain the nuclei of migrated cells for visualization. The migrated cells were then counted with a fluorescence microscope.

### 2.6. Cell Transfection

For siRNA transfection, NPC cells were transfected with siRNA or miRNA as previously described [27,28]. In brief, fibronectin (10 μg/mL) was pre-coated on 35 mm culture plates at 4 °C for 24 h. NPC cells (3 × 10^5^) were seeded in fibronectin pre-coated plates for 24 h before transfection. Lipofectamine^®^ 2000 (Invitrogen, Thermo Fisher Scientific, Waltham, MA, USA) was used in all transient transfection studies, following the manufacturer’s instructions. In the siRNA knockdown experiment, cells were transfected with 50 nM ON-TARGETplus SMARTpool Human *ITGB1* siRNA (Dharmacon, Lafayette, CO, USA; Cat. ID L-004506-00-0005). For scramble control, ON-TARGETplus siCONTROL Non-Targeting siRNA (Dharmacon; Cat. ID D-001810-01-20) was used.

The transfection of precursor miRNA (pre-miRNA) was performed as previously described [29]. Briefly, the cells were transfected with 50 nM pre-miR-134 (Ambion; P/N: AM17100; ID: AM20391) for 48 h with Lipofectamin 2000. The miRNA precursor negative control (Pre-control, ID: AM17110) was obtained from Ambion. After transfection, the cells were collected for the subsequent analysis.

For stable expression of miR-134 in NPC cells, the pLVX expression vector was used [37]. The packaging vectors psPAX2 and pMD2.G were obtained from Addgene (Watertown, MA, USA). The pLVX-EF1α-miR-134 precursor-expressing vector was constructed. Lentivirus packaging was performed using 293T cells and X-tremeGENE transfection reagent (Millipore, # 6366236001, Burlington, MA, USA). The 293T cells were incubated for 48 h for virus production. Subsequently, the lentivirus-containing medium was harvested and stored in a −80 °C freezer until usage. For infection, NPC cells resuspended in fresh complete medium were mixed with the lentivirus-containing medium at a ratio of 1:1 in a culture flask, with the addition of polybrene (Santa Cruz Biotechnology, # sc-134220, Dallas, Texas, USA). After 12 h, the medium was replaced with fresh complete medium. Successfully infected cells were selected by antibiotics Puromycin (Invivogen, # ant-pr-1, San Diego, CA, USA).

### 2.7. In Vitro Inverted Matrigel Culture

The in vitro inverted Matrigel assay was adapted from a previous study [38]. A total of 30,000 cells mixed in 50 μL of Matrigel (Merck KGaA, #354230, Darmstadt, Germany) were seeded into a culture-insert (SPL #35224, Pocheon-si, South Korea) and the gel mixture was allowed to solidify. A second layer of 200 μL Matrigel was then layered on top of the first layer of cell-embedding Matrigel. For ICG-001 treatment, cells were cultured in DMEM/F12 (Gibco, #11320033, Waltham, MA, USA) supplemented with 20 ng/mL EGF, 20 ng/mL FGF, 20 ng/mL IGF for one week before starting the drug treatment. During drug treatment, the lower chamber is filled with 200 μL of 10 μM ICG-001 or DMSO dissolved in plain RPMI, while the culture insert is filled with 200 μL of 10 μM ICG-001 or DMSO dissolved in RPMI supplemented with 20% FBS and 50 ng/mL EGF for chemoattraction. The culture medium was replaced every two days during incubation. The cells were cultured for another two weeks and then imaged with a confocal microscope with a z-stack analysis. For transient transfection of miR-134, the cells were transfected with pre-miR-134 or pre-miR-Ctrl, as described in Section 2.6. The cells were cultured in a similar manner for one week before using plain RPMI in the lower chamber and RPMI supplemented with 20% FBS and 50 ng/mL EGF in the culture insert for chemoattraction. The cells were then incubated for two weeks before imaging. Total fluorescence signal of spheroids migrated longer than 200 μm was divided by total fluorescence signals of all spheroids to obtain the percentage of spheroids with long migrating distance.

### 2.8. 3′-UTR Luciferase Reporter Assay

To study the interaction between miRNA-134 and the 3′-UTR of *ITGB1* mRNA, the miRNA-134 binding sequence in wild-type *ITGB1* mRNA 3′-UTR (AGTAGCAATTTCCATAGTCACAGGAATTCATCTTGTTTCACACTAGTCACAT), a target-site mutant (AGTAGCAATTTCCATAACTGCAGGAATTCATCTTGTTTCACACTAACTGCAT), and a miRNA-134 sensor sequence (CCCCTCTGGTCAACCAGTCACAGAATTCCCCCTCTGGTCAACCAGTCACA) were cloned individually downstream to the firefly luciferase of dual luciferase reporter plasmid pmirGLO (Promega, Madison, WI, USA), according to the manufacturer’s instruction. The sequences were based on a previous publication [39]. Plasmids and pre-miR-134 were co-transfected into HONE-1 cells using Lipofectamine 2000 for 48 h. The luciferase activity was determined using the Dual-Luciferase reporter assay kit (Promega, Madison, WI, USA) with a microplate luminometer and Firefly luciferase activity was normalized to Renilla luciferase activity.

### 2.9. Western Blotting

Western blotting was carried out as previously described [40]. NPC cells with various treatments were lysed by lysis buffer containing 1% of NP40, 250 mM of Tris, 150 mM of NaCl, 0.25% of protease inhibitor, and 1% of phosphatase inhibitor. The lysates were then centrifuged at 14,000 rpm for 15 min at 4 °C to get rid of undissolved cell debris. DC Protein Assay Kit (Bio-rad, Hercules, CA, USA) was used for the protein concentration determination. For protein denaturation and unfolding, samples were boiled with SDS containing buffer for 8–15 min. Samples were stored in a −20 °C freezer until usage. For the Western blotting, protein samples of equal amount were resolved by 8% SDS-polyacrylamide gels. The resolved protein samples were then transferred to PVDF membranes (Millipore, Burlington, MA, USA). The membrane was then blocked by 5% of non-fat milk for 1 h at room temperature. After that, the primary antibody against *ITGB1* (Cell Signaling Technology, #9699, Danvers, MA, USA) was applied and incubated for 2 h at room temperature with gentle shaking. After washing with 5% TBST, HRP-conjugated secondary antibody and substrates were applied. The protein bands were detected by a Chemidoc gel imaging system (Bio-Rad, Hercules, CA, USA) or by X-ray films and analyzed by Image J.

### 2.10. Confocal Microscopy

C666-1, HONE-1, and HK-1 cells were initially grown on cover slips. The coverslips were then transferred to 35 mm culture dishes and treated with ICG-001 for 7 days. Then, the medium was discarded and PBS was used to rinse the cells for three times. Cells were fixed with 4% paraformaldehyde for 10 min. The cell-containing 35 mm dishes were then placed on an ice bath and incubated with primary and secondary antibodies. Afterwards, the cells were fixed with a DAPI-containing Mountant (ProLong Gold Antifade Mountant, Thermofisher, #P36930). The confocal images of cells stained with DAPI, integrin α5β1 (antibody from Millipore, Clone JBS5, #MAB1969) with AlexaFluor-488 conjugated donkey anti-mouse secondary antibody (Invitrogen, #A21202), and Phalloidin (antibody from ThermoFisher, #A12381) were captured under confocal microscopy at an excitation wavelength of 405, 495, and 595 nm, respectively.

### 2.11. In Vivo Lung Metastasis Assay

The in vivo lung metastasis assay was conducted on female athymic BALB/c/nu/nu nude mice (6–8 weeks old). The mice were supplied by the Laboratory of Animal Unit of the University of Hong Kong and were housed in the Hong Kong Baptist University animal house. The mice were kept in sterile systems with independent ventilation. Sufficient sterile water and food were provided for the mice. The luciferase labeled NPC HONE-1 cells (HONE-1-luc) were first pre-treated with ICG-001 or solvent control to study the effects of ICG-001 on the metastasis of NPC cells. To study the role of miR-134 in the metastasis of NPC cells, HONE-1-luc cells were pre-transfected with transient/stable miR-134 precursor or corresponding negative control miRNA. Cells were then collected, resuspended in plain medium, and introduced into the mice through i.v. injection at a cell number of 1 × 10^6^ per nude mouse. After 28 days, the mice were anesthetized and luciferase substrate was injected subcutaneously for the imaging. Imaging of the real-time bioluminescence signals emitted from the tumor cells was conducted with IVIS Lumina XR Small Animal Imaging System. The lungs were fixed with formalin, embedded in paraffin, and histological sections were stained with hematoxylin and eosin (H&E) according to the general practice. The infiltrated metastatic tumor cells in the lungs were then identified to validate the bioluminescence signals. The animal study was approved by Hong Kong Baptist University Research Ethics Committee (Ref. #: HASC/16-17/0208).

### 2.12. Statistical Analysis

Data are presented as the mean ± standard deviation of ≥3 independent experiments. The difference between control and treatment groups was determined by Student’s *t*-test using IBM SPSS version 22.0. A *p* < 0.05 was considered to indicate a statistically significant difference.

## 3. Results

### 3.1. ICG-001 Reduces NPC Cell Adhesion Capability

**Figure 1:**
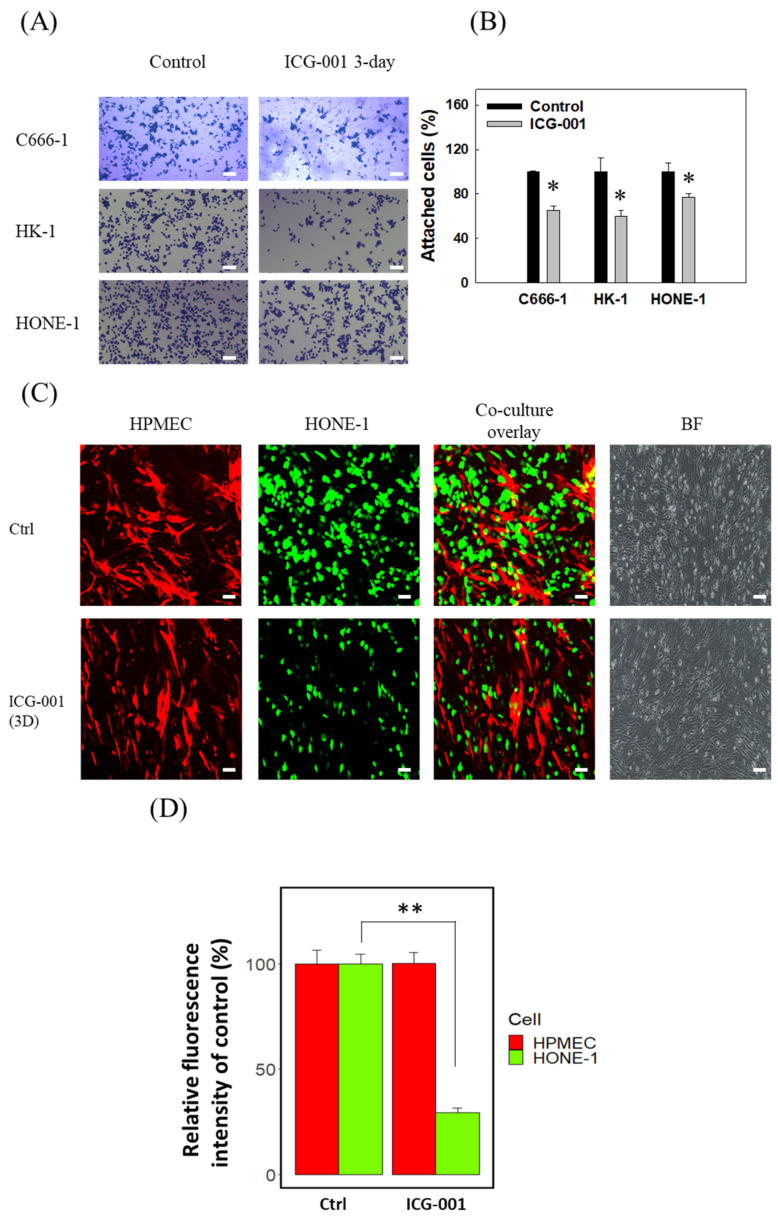
The Wnt/CBP/β-catenin antagonist ICG-001 suppresses the cell–matrix and tumor cell–endothelial cell adhesion of NPC cells. (**A**) ICG-001 (10 μM, 3-day treatment) inhibited the adhesion of C666-1, HK-1, HONE-1 cells to fibronectin-coated 96-well plate. Scale bar, 40 μm. (**B**) Statistical summary of inhibitory effects of ICG-001 on adhesion of NPC cells. (**C**) ICG-001 (3-day treatment) inhibited the adhesion of HONE-1 cells to HPMEC. Scale bar, 20 μm. (**D**) Statistical summary of inhibitory effects of ICG-001 on adhesion of NPC HONE-1 cell to HPMEC. Results were obtained from three independent experiments. * *p* < 0.05, ** *p* < 0.01.

To investigate if ICG-001 could serve as a therapeutic drug for suppression of NPC metastasis, we tested if ICG-001 could suppress cell–cell and cell–extracellular matrix adhesion capability. Two types of adhesion experiments were conducted. Firstly, NPC cells with/without ICG-001 pre-treatment were allowed to attach to fibronectin-coated 96-well tissue culture plates (Corning^TM^ Falcon^TM^, #353072, Tewksbury, MA, USA). As shown in Figure 1A,B, treatment with ICG-001 significantly reduces the number of cells attached in NPC C666-1, HONE-1, and HK-1 cells. Next, we studied the adhesion capability of HONE-1 cells to the Human Pulmonary Microvascular Endothelial Cells (HPMEC) to mimic lung adherence, as the lung ranks as the third most common metastatic site of NPC [41]. As shown in Figure 1C,D, ICG-001 treatment reduced the adherence of HONE-1 cells to HPMEC cells by 70%. Our previous work showed that ICG-001 treatment reduces NPC cell migration capability, as demonstrated by a transwell migration assay [28,29]. We also showed that ICG-001 inhibits the migration of C666-1 cells with a wound healing assay (Figure S1). Together, these data showed that ICG-001 could suppress the migration and cell–matrix and tumor cell–endothelial cell adhesion capability of NPC cells.

### 3.2. ICG-001 Suppresses NPC Adhesion and Migration via Upregulating the Expression of miR-134

**Figure 2:**
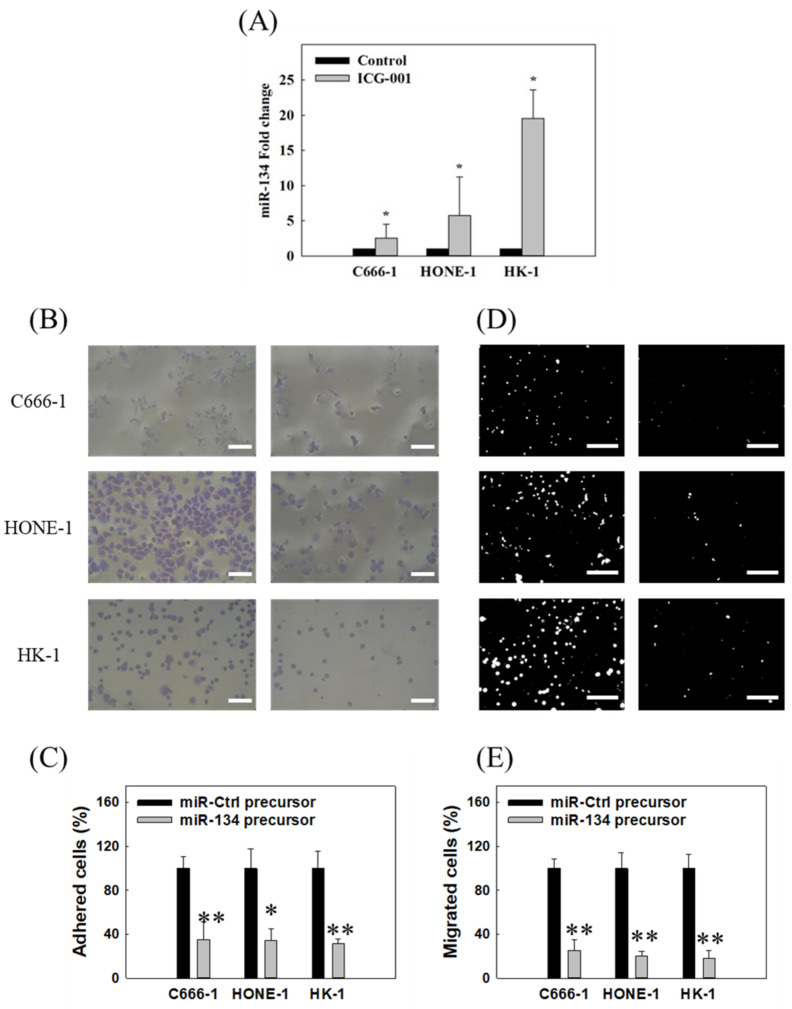
ICG-001 inhibits NPC adhesion and migration via upregulating the expression of miR-134. (**A**) ICG-001 treatment increased expression of miR-134 in three NPC cell lines. qPCR was used to study the miRNA expression. Control, solvent control. (**B**) miR-134 overexpression inhibited adhesion of NPC cells. Scale bar, 40 μm. (**C**) Statistical summaries of the inhibitory effects on NPC adhesion from three independent experiments. (**D**) miR-134 overexpression inhibited migration of NPC cells. Scale bar, 100 μm. Extracellular matrix adhesion assay was used to study the tumor cell–fibronectin adhesion as described in Figure 1A. A transwell migration assay was used to analyze the tumor cell migration. (**E**) Statistical summaries of the inhibitory effects on NPC migration from three independent experiments. * *p* < 0.05, ** *p* < 0.01.

To identify novel targets that contribute to the ICG-001-mediated suppression of NPC migration and adhesion, a list of EMT and CSC-related miRNAs was screened using C666-1 and HONE-1 cells grown in both monolayer culture and CSC-enriched tumor sphere culture. Results (Figure S2) showed that miR-134 was consistently upregulated by ICG-001 treatment. Such upregulation was further validated in C666-1, HK-1, and HONE-1 cells by PCR analysis (Figure 2A). Little is known regarding the function of miR-134 in NPC. By transient transfection of miR-134 precursor, it was found that miR-134 reduced the adhesion capability of NPC C666-1, HONE-1, and HK-1 cells by around 60% (Figure 2B,C). In addition, the transient miR-134 precursor expression could reduce migration capability by around 70% in the three NPC cell lines (Figure 2D,E). On the other hand, it was also found that miR-134 did not significantly affect the growth of CSC-enriched NPC tumor spheres (Figure S3).

**Figure 3:**
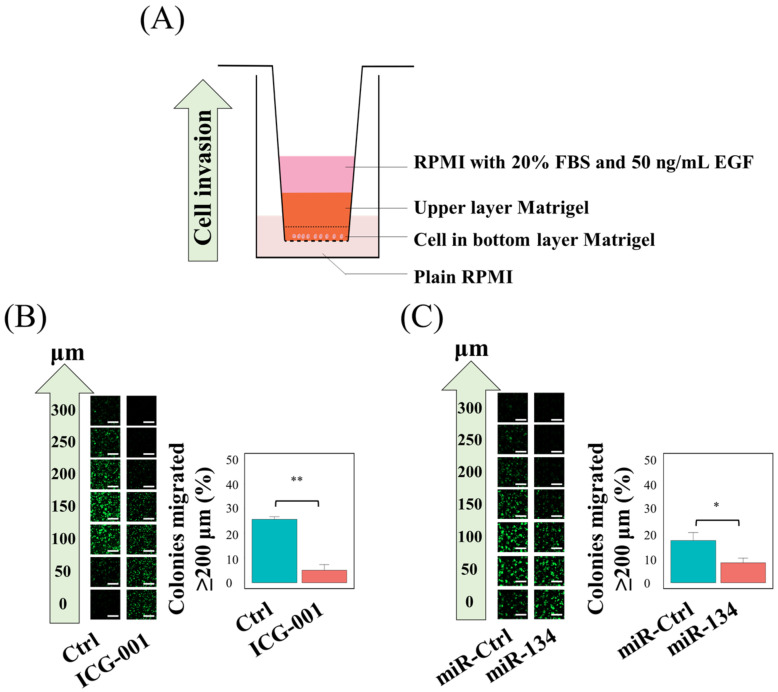
ICG-001 and miR-134 inhibit NPC invasion in a 3D Matrigel culture model. (**A**) A schematic illustration of the 3D Matrigel culture. NPC cells were seeded into the bottom layer Matrigel. This first layer of Matrigel was allowed to solidify before layering a second layer of Matrigel on top of it. Cells were allowed to grow for one week for spheroid formation before imposing a chemo-gradient for spheroid invasion towards the upper layer Matrigel. Both (**B**) ICG-001 and (**C**) transient overexpression of miR-134 could inhibit the invasion of NPC C666-1 spheroids. Results were shown from three independent experiments. Scale bar in (**B**) and (**C**), 500 μm. * *p* < 0.05; ** *p* < 0.01.

To further test the roles of ICG-001 and miR-134 on the adhesion/migration/invasion capability of NPC cells, an in vitro inverted three-dimensional (3D) Matrigel culture model was adopted. A schematic illustration of the 3D Matrigel culture was shown in Figure 3A. Results showed that both ICG-001 (Figure 3B) and transient overexpression of miR-134 (Figure 3C) could reduce the percentage of spheroids with long invasion distance. Taken together, these data showed that miR-134 functions mainly to reduce cell adhesion and migration in NPC cells, but not CSC growth.

### 3.3. miR-134 Targets ITGB1 in NPC

**Figure 4:**
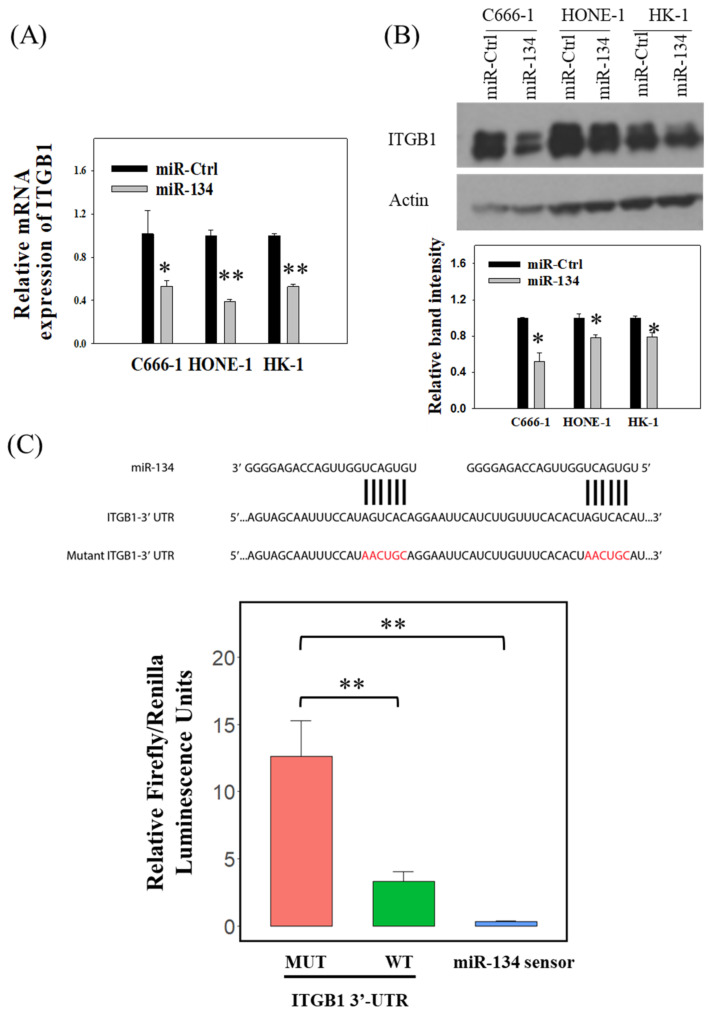
miR-134 targets *ITGB1* in NPC. (**A**)Transient overexpression of miR-134 in NPC cells reduced the expression of *ITGB1* mRNA. (**B**) Overexpression of miR-134 in NPC cells reduced the expression of *ITGB1* protein. The protein expression was detected by Western blot analysis, and actin serves as a loading control. Statistical summary from three independent experiments was also shown. (**C**) miR-134 specifically targeted *ITGB1* in NPC. The sequences of wild-type and mutant *ITGB1* 3′-UTR and the base-paring between miR-134 and *ITGB1* 3′-UTR were indicated in the top panel; the specific inhibition of miR-134 on the wild type *ITGB1* 3′-UTR containing luciferase activity was displayed in the bottom panel. MUT, mutant; WT, wild type. * *p* < 0.05, ** *p* < 0.01.

Although less studied in NPC, the role of miR-134 has been studied in other cancer models. It has been previously reported that miR-134, through targeting *ITGB1*, suppressed metastasis of hepatocellular carcinoma [42]. In mesenchymal stem cells, negative regulation of *ITGB1* by miR-134 resulted in reduced cell adhesion [43]. It is therefore possible that miR-134 exerts its suppression on tumor adhesion and migration in NPC via downregulation of *ITGB1*. Transient transfection of miR-134 precursor efficiently downregulated the expression of *ITGB1* mRNA (Figure 4A) and protein (Figure 4B, whole blot is shown in Figure S4A) in NPC cells. Using a 3′-UTR reporter assay, it was demonstrated that miR-134 specifically reduced luciferase activity when the wild-type 3′-UTR region of ITGB1 was included in the promoter region of the luciferase, but not affecting the luciferase activity, when the mutant 3′-UTR region of ITGB1 was included (Figure 4C).

RNA sequencing was conducted for NPC C666-1 cells with forced miR-134 expression so as to determine whether this miRNA has other target genes besides *ITGB1*. The efficiency of miR-134 overexpression is shown in Figure S5A and 1860 genes were identified to be differentially expressed after miR-134 overexpression (Figure S5B). Furthermore, when the microRNA target prediction tool “PicTar” (https://pictar.mdc-berlin.de/, accessed on 10 June 2022) was used to predict the miR-134 target genes, *ITGB1* was found to be one of the top five predicted target genes (Table S1). Interestingly, when including both PicTar and miRDB prediction programs (http://mirdb.org/, accessed on 10 June 2022), only one predicted target gene overlaps with the 1118 significantly downregulated genes shown in the RNA sequencing data (Figure S5C and Table S2), suggesting that most of these genes seem to be indirectly affected by miR-134. Indeed, the RNA sequencing results showed that *ITGB1* expression in miR-134 transfected C666-1 was 0.52 times that of the miR-Ctrl (Table S3), which is comparable to our qPCR results (Figure 4A). Gene ontology (GO) enrichment analysis showed that the downregulated genes were most significantly associated with perception of taste and modulation of receptor activity (Figure S5D). Kyoto Encyclopedia of genes and genomes (KEGG) enrichment analysis showed that none of the metabolism-related KEGG terms were significantly associated with the down-regulated genes by miR-134 overexpression (Figure S5E). Taken together, the RNA sequencing data, the target gene prediction analysis, and the reporter assay results suggested that *ITGB1* is one of the best candidate target genes regulated by miR-134 in EBV-associated NPC cells.

### 3.4. ITGB1 Is Involved in the Reduced Cell Adhesion and Migration Capacity by ICG-001 and miR-134

**Figure 5:**
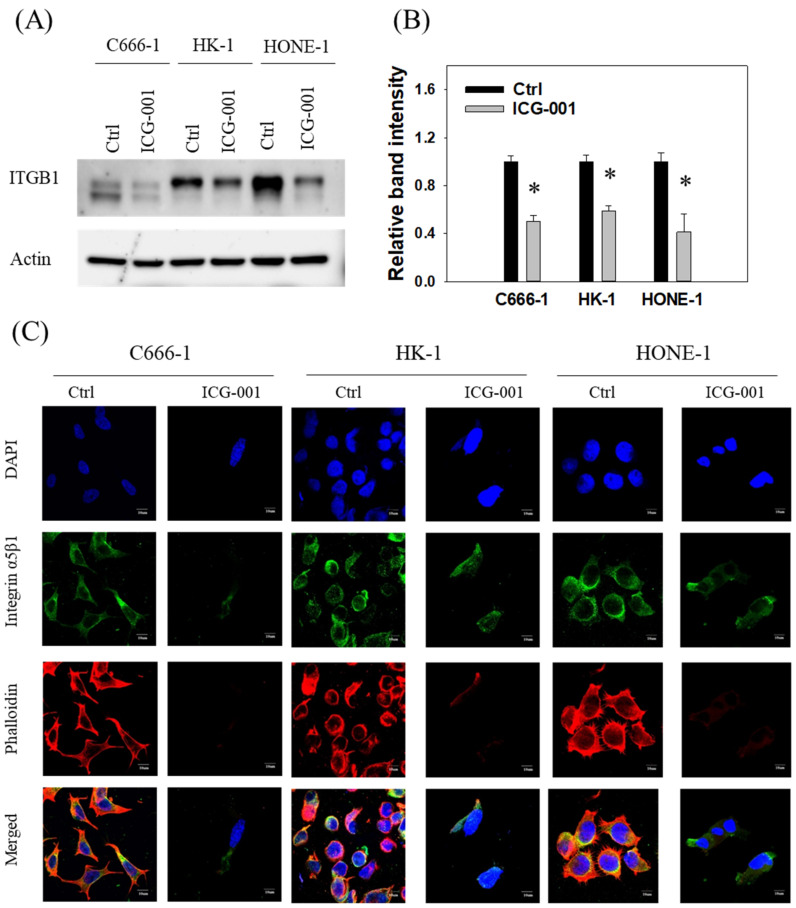
ICG-001 downregulates the expression of *ITGB1* in NPC cells. (**A**) ICG-001 treatment (10 μM, 3-day) inhibited the expression of *ITGB1* in NPC cells. The *ITGB1* protein expression was detected by Western blot analysis, and actin serves as a loading control. A statistical summary is displayed in (**B**). (**C**) ICG-001 treatment (10 μM, 7-day) reduced the expression of integrin α5β1 and phalloidin in NPC cells. The expression of integrin α5β1 and phalloidin was detected by immunofluorescent imaging. * *p* < 0.05.

The expression of *ITGB1* has been found in several studies to be upregulated in NPC and contributes to NPC migration and invasion [44,45,46]. Given that ICG-001 induced upregulation of miR-134 and that *ITGB1* is a direct target of miR-134, we next studied the effects of ICG-001 treatment on the expression of *ITGB1*. As shown in Figure 5A,B (whole blot included in Figure S4B), ICG-001 treatment could reduce the expression of *ITGB1* in NPC C666-1, HK-1, and HONE-1 cells. Quantitative analysis of the protein band intensity showed that the expression of *ITGB1* was decreased by 40–50% in those three cell lines. Functional integrins are heterodimers that consist of α and β subunits. Previous study showed that integrin α5β1 plays an important role in the migration of nasal epithelial cells, especially during wound repairing [47]. As integrin α5β1 has been reported to be upregulated by EBV oncoprotein LMP1 [46], we studied the expression of integrin α5β1 by confocal microscopy. Results showed that the expression of integrin α5β1 was decreased by ICG-001 treatment (Figure 5C). The decreased expression of integrin α5β1 affected the formation of actin filaments, as phalloidin expression was also decreased by ICG-001 treatment.

**Figure 6:**
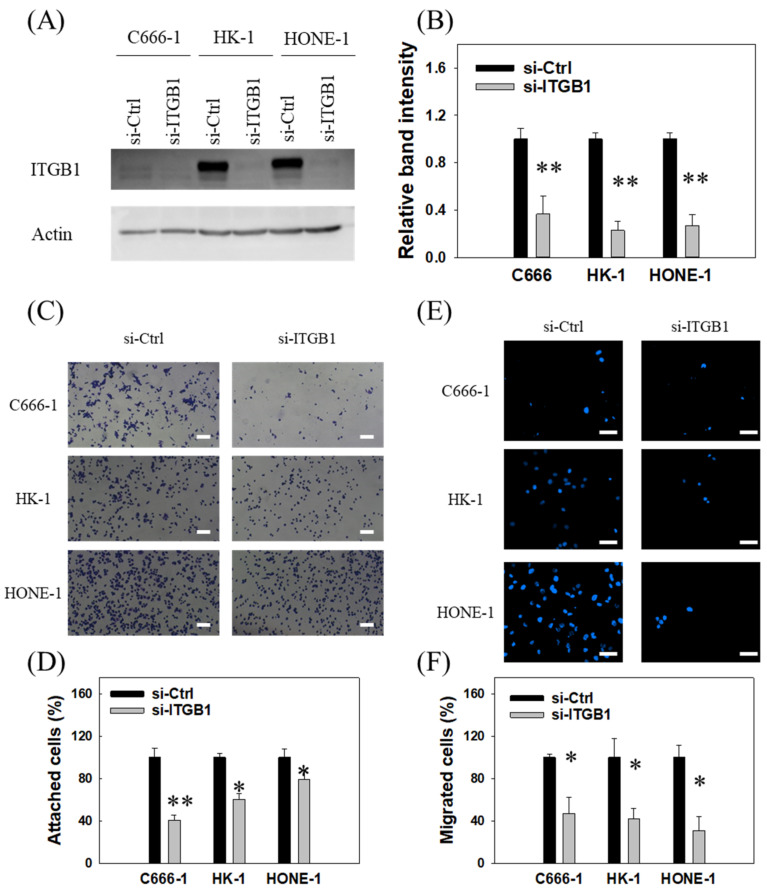
*ITGB1* is involved in the suppression of tumor cell adhesion and migration of NPC cells by ICG-001. (**A**) Effects of *ITGB1* siRNA on the expression of *ITGB1* in NPC cells. The protein expression was detected by Western blot analysis, and actin serves as a loading control. si-Ctrl, scramble siRNA control. (**B**) Statistical summary of knockdown efficiency from three independent experiments. (**C**) *ITGB1* siRNA inhibited adhesion of NPC cells. Extracellular matrix adhesion assay was used to study the tumor cell-matrix adhesion as described in Figure 1A. Scale bar, 40 μm. (**D**) Statistical summaries of the effects of *ITGB1* knockdown on cell adhesion from three independent experiments. * *p* < 0.05, ** *p* < 0.01. (**E**) *ITGB1* siRNA inhibited migration of NPC cells; a transwell migration assay was used. Scale bar, 50 μm. (**F**) Statistical summaries of the effects of *ITGB1* knockdown on tumor cell migration from three independent experiments. * *p* < 0.05, ** *p* < 0.01.

To examine if downregulated *ITGB1* expression could contribute to ICG-001-mediated suppression of NPC adhesion and migration, knockdown of *ITGB1* was conducted via transient transfection with siRNA. As shown in Figure 6A,B (whole blot included in Figure S4C), the *ITGB1* siRNA led to 60–70% reduction in the expression levels of *ITGB1* in C666-1, HK-1, and HONE-1 cells. Reduction of *ITGB1* by siRNA treatment led to 20–60% reduction in the adhesion capability (Figure 6C,D), and 60–70% reduction in the migration capability of NPC cells (Figure 6E,F). Taken together, these data showed that the reduced adhesion and migration capacity of NPC cells mediated by ICG-001 was due, at least partially, to the miR-134/*ITGB1* axis.

### 3.5. ICG-001 Suppresses the Metastasis of NPC in the Nude Mice

**Figure 7:**
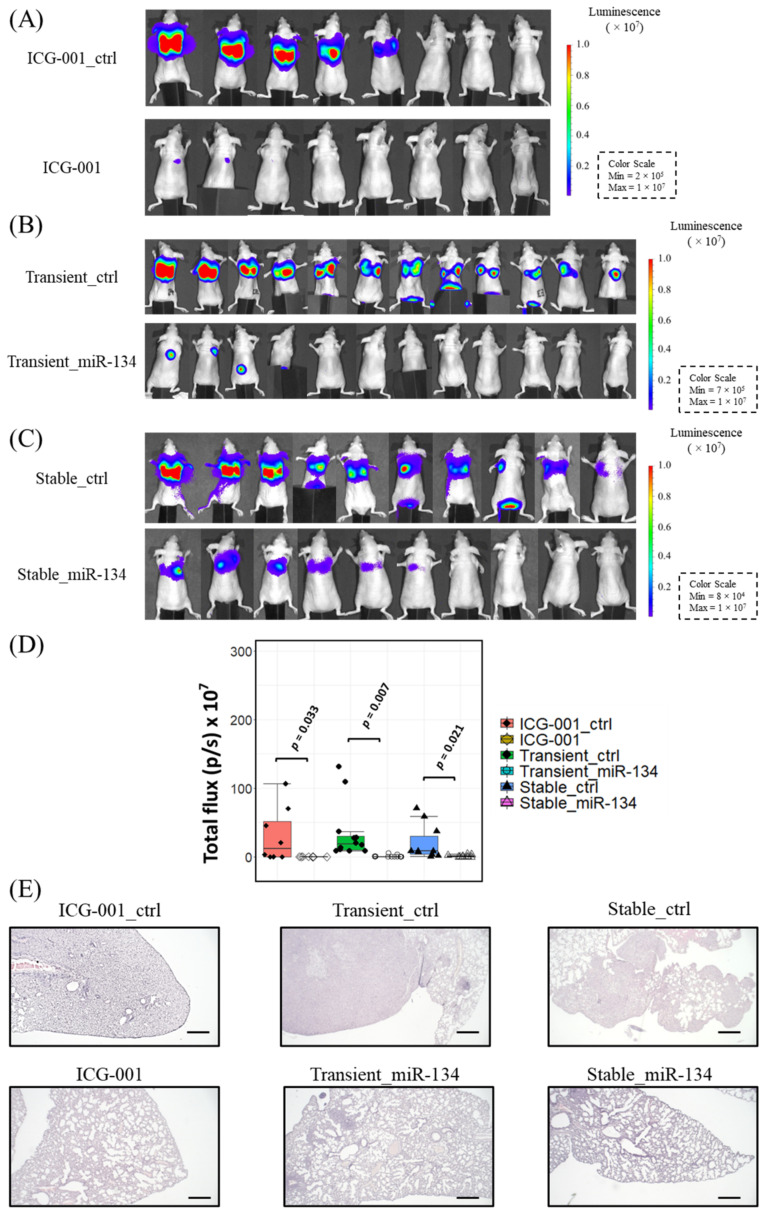
Inhibitory effects of ICG-001 treatment and the upregulated miR-134 expression on lung metastasis of NPC cells. (**A**) The ICG-001 pre-treatment (10 μM, 7 days) inhibited the metastasis of HONE-1-luc cells in nude mice (*n* = 8). (**B**) Transient overexpression of miR-134 inhibited the metastasis of HONE-1-luc cells in nude mice (*n* = 12). (**C**) Stable overexpression of miR-134 inhibited the metastasis of HONE-1-luc cells in nude mice (*n* = 10). (**D**) Statistical summary of the suppressive effects of ICG-001, transient/stable miR-134 transfection on the metastasis of NPC cells to the lung of mice. (**E**) Representative H&E staining of the mice lung. (Scale bar, 2 mm).

To demonstrate the effect of ICG-001/miR-134/*ITGB1* axis on the metastasis of NPC cells in vivo, a luciferase reporter HONE-1 cell line (HONE-1-luc) was generated. The cells were treated with ICG-001 or solvent control, and then injected to nude mice through intravenous (i.v.) injection. As shown in Figure 7A, the bioluminescence signals from ICG-001 pre-treated HONE-1-luc were drastically reduced when compared to the solvent control group. The average photon flux was 270-fold higher in the control group mice than in the treatment group (*n* = 8). Transient transfection of miR-134 precursor into HONE-1-luc cells led to an enormous reduction in the metastasized HONE-1-luc cells. As shown in Figure 7B, the average photon flux was 40-fold higher in the control group mice than in the treatment group (*n* = 12). Stable transfection of miR-134 precursor resulted in similar suppression of metastasis (Figure 7C), where average photon efflux was 20-fold higher in the control group (*n* = 10). Statistical data are summarized in Figure 7D. H&E staining of mice lungs (Figure 7E) was used to validate the bioluminescence results and showed that in the control group, lung infiltration of NPC cancer cells was much more severe than in the treatment groups. Immunohistochemical (IHC) staining of the lung infiltrated with NPC cells for one functional integrin heterodimer, integrin α5β1, confirmed that the expression of this heterodimer was downregulated by ICG-001/miR-134 (Figure S6). Taken together, it can be seen that the ICG-001 pre-treatment could efficiently suppress the metastasis of NPC cells in this animal model, and such suppression was at least partially due to the upregulation of miR-134 in NPC cells.

## 4. Discussion

Canonical Wnt/β-catenin signaling is frequently hyperactivated through mechanisms such as epigenetic silencing of negative Wnt regulators [18,48], phosphorylated inactivation of Glycogen Synthase Kinase 3 Beta (*GSK3β*) [49], and the high level expression of the β-catenin coactivator CBP [17,50]. It is therefore possible to control the progression of NPC by antagonizing β-catenin/CBP binding with ICG-001. In this study, we found that the CBP/β-catenin antagonist ICG-001 could inhibit cell–matrix adhesion and tumor cell–endothelial cell adhesion (Figure 1). Previously we have reported that ICG-001 treatment can reduce the migration capability of NPC cells [28,29].

There is a complex interplay between miRNAs and Wnt/β-catenin signaling. To date, more than 30 different miRNAs have been found to target components of the Wnt/β-catenin signaling pathway, while more than ten different miRNAs have been reported to be targeted by the Wnt/β-catenin signaling pathway [26,51]. As aberrant expression of both miRNAs and Wnt/β-catenin signaling molecules has been well-documented in different cancer types, those molecules could serve as therapeutic targets. A recent study showed that a Wnt inhibitor, NCB-0846, could increase the expression of miRNAs targeting the TGFβ receptor type-I (*TGFBR1*) gene, inhibit the EMT induction by TGFβ, and abolish the lung metastasis of TGFβ1-treated A549 cells [52]. We found that the expression of miR-134 was regulated by ICG-001 (Figure 2A) and that upregulation of miR-134 could inhibit the migration and adhesion capacity of NPC cells (Figure 2B,D). Furthermore, using a 3D Matrigel culture model, we demonstrated that both ICG-001 and miR-134 could inhibit the invasion of NPC spheroids (Figure 3B,C). This is a novel finding that expression of miR-134 can be regulated by the Wnt/CBP/β-catenin pathway. This finding is in concordance with some previous studies: (1) elevated level of tumor necrosis factor alpha (*TNFα*) is unfavorable for the survival of NPC patients [53], (2) *TNFα* treatment was shown to increase the expression of the β-catenin protein in nucleus pulposus cells [54], and (3) TNFα has been recently shown to suppress the expression of miR-134-5p in human airway epithelial cells [55]. It is possible that the elevated level of TNFα in NPC might lead to hyperactivation of the Wnt/CBP/β-catenin pathway which could suppress the miR-134 expression, and the miR-134 expression can be restored by switching to the Wnt/p300/β-catenin pathway with ICG-001. In addition, we clearly showed that the miR-134 expression could decrease the expression of *ITGB1* in NPC cells (Figure 4A,B). Using a 3′-UTR luciferase assay, we confirmed that *ITGB1* is a direct target of miR-134 (Figure 4C). These findings can provide a functional explanation for previous published studies in which low miR-134 expression levels were observed in NPC patients [31,32]. On the other hand, as shown by the RNA sequencing results (Table S3) and the miRNA target gene prediction analysis (Table S1), *ITGB1* was shown to be the best candidate target gene of miR-134. However, we cannot preclude that miR-134 might have other target mRNAs in NPC cells, including mRNAs that were enriched in the gene sets affecting the receptor–ligand activity. In this study, we have verified the importance of the miR-134/*ITGB1* axis in modulation of NPC metastasis; verification of other candidate miR-134 targets in NPC remains to be explored by future studies.

Expression of integrin α5β1 has been reported to be dysregulated in many cancers. In melanocytic tumors, integrin α5β1 was found to be expressed, while such expression was not seen in the non-tumor counterpart [56]. In ovarian cancer, high expression of integrin α5β1 was found to correlate with more advanced clinical stage [57]. Upregulation of integrin α5β1 correlated with poor prognosis and tumor progression in lung cancer [58]. In NPC, the expression of EBV oncoprotein LMP1 has been reported to upregulate expression of integrin α5β1 [46]. Previously, it was found that both expression and activation of integrin α5β1 could activate the transcription of β-catenin target genes in glioma cells [59]. Our observation that blocking the CBP/β-catenin interaction could lead to downregulated integrin α5β1 expression (Figure 5C) suggested that there may be a positive feedback loop between expression of integrin α5β1 and Wnt/β-catenin signaling, culminating in an aggressive phenotype observed in metastatic NPC.

The observation that F-actin was also downregulated by ICG-001 treatment is worthy of discussion. Integrins bind to their extracellular ligands via their outer domains and serve as traction points for contractile forces through their interaction with actin. At the same time, integrin signaling activates Rho GTPases, which actively control the polymerization and organization of actin [60]. *ITGB1* plays a critical role in development of lens fiber cells, where lens of *ITGB1* knockout mice had profound defects in fiber cell morphology, associated with the loss of the F-actin network [61]. It has been recently reported that gingipains led to the disruption of the F-actin network in human calvarial osteoblasts, and such an effect was mediated by *ITGB1* [62]. In that study, knockdown of *ITGB1* resulted in disruption of the F-actin network. However, the functional role of F-actin expression in cancer metastasis is not clear-cut. In a study including 210 breast cancer tissues, IHC staining showed that F-actin expression is significantly higher than in normal breast tissue [63]. In lung cancer, tropomyosin 4 has been reported to promote cell migration and such effects were due to increased F-actin assembly with tropomyosin 4 overexpression [64]. Yet, some researchers found that lower phalloidin expression was linked to higher metastatic potential in osteosarcoma cells [65]. Our observation that phalloidin expression was disrupted by ICG-001 and such disruption was associated with reduced migration capability of NPC cells, suggested a pro-metastatic role of phalloidin in NPC.

**Figure 8:**
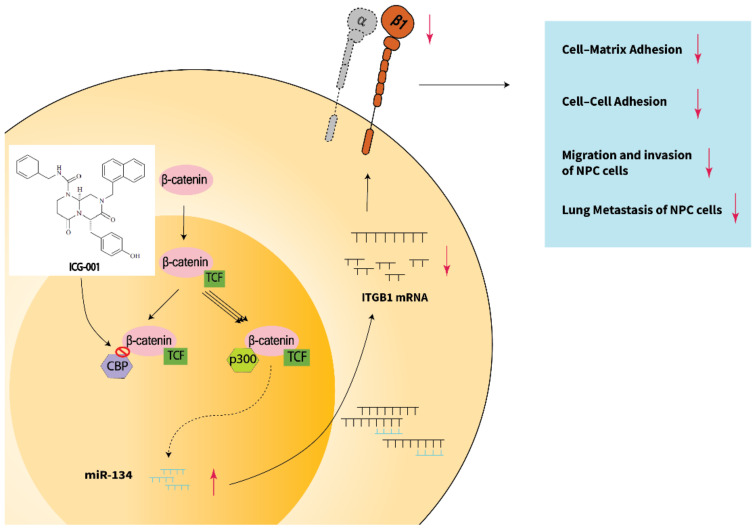
ICG-001 inhibits NPC tumor metastasis via the miR-134/*ITGB1* axis. A β-catenin/TCF complex in the nucleus could either bind with CBP or p300. The CBP-bound form favors cell proliferation while the p300-bound form favors cell differentiation. ICG-001, via blocking the binding between β-catenin and CBP, favors the formation of the p300/β-catenin/TCF complex. As a result, the miR-134 expression is upregulated by ICG-001 and that subsequently mediates downregulation of *ITGB1*. Reduced expression of *ITGB1*, including the functional heterodimer of *ITGB1* with an alpha subunit (e.g., Integrin α5β1), can result in reduced cell–matrix adhesion, cell–cell adhesion, cell migration and invasion, and lung metastasis of NPC cells.

The in vitro and in vivo overexpression of miR-134 study (Figure 2 and Figure 7) demonstrate that miR-134 is a potent inhibitor of NPC metastasis. As metastatic NPC remains incurable [66] and distant metastasis remains the dominant cause of treatment failure in NPC [5], therapeutic intervention to restrict NPC metastasis is critical for improving the NPC treatment outcome. Our discovery that modulation of the Wnt/β-catenin signaling pathway via the miR-134/*ITGB1* axis resulted in a drastic reduction in distant metastasis, suggested that therapeutic targeting of the Wnt/β-catenin pathway is likely a promising approach for controlling NPC metastasis. A schematic summarization of the main findings of this study is presented in Figure 8.

## 5. Conclusions

In this study, the anti-metastatic function of the CBP/β-catenin antagonist, ICG-001, in NPC was examined. The EMT-related miR-134 was identified as a target modulated by ICG-001 by initial screening followed with qPCR verification in a panel of NPC cell lines. Down-regulated miR-134 expression was reported to be negatively associated with NPC prognosis [31,32], yet the underlying mechanisms remained a mystery. We demonstrated that miR-134 directly targets ITGB1 to inhibit the expression of ITGB1, thereby reducing the adhesion, migration, and invasion capability of the NPC cells. The anti-metastatic function of ICG-001 and miR-134 was further demonstrated with the in vivo lung metastasis model. With cancer metastasis being the main cause of treatment failure for NPC, our study shed light on the opportunity of modulating the Wnt signaling pathway for battling with NPC.

## Data Availability

All data are included in the main text or in the supplementary files.

## Supplementary Materials

The following supporting information can be downloaded at: https://www.mdpi.com/article/10.3390/cancers14133125/s1, Figure S1: ICG-001 inhibits the wound healing of C666-1 cells; Figure S2: A screening on EMT and CSC related miRNAs identifies miR-134 as a target of ICG-001; Figure S3: miR-134 does not affect the growth of CSCs-enriched NPC tumor spheres; Figure S4: The whole blots of western blot data; Figure S5: miR-134 is involved in modulation of receptors and taste sensory in NPC; Figure S6: Expression of integrin α5β1 in lung-infiltrating NPC tumor cells; Table S1: PicTar predicts *ITGB1* as top 5 target of miR-134; Table S2: Comparison of down-regulated genes from RNA sequencing with software-predicted miR-134 targets; Table S3: RNA sequencing result of miR-134 overexpression in C666-1 cells [67,68,69,70].
