# Supplementary material for: The CBP/β-Catenin Antagonist, ICG-001, Inhibits Tumor Metastasis via Blocking of the miR-134/ITGB1 Axis-Mediated Cell Adhesion in Nasopharyngeal Carcinoma
Source: Cancers (Basel). 2022 Jun 25;14(13):3125. doi: 10.3390/cancers14133125 (PMC9264930; doi:10.3390/cancers14133125)
Supplement: Supplementary file 1 [file cancers-14-03125-s001.zip › cancers-1581064-supplementary.pdf]

cancers

MDPI

Supplementary Materials

The CBP/β-Catenin Antagonist, ICG-001, Inhibits Tumor Metastasis via Blocking of the miR-134/ITGB1 Axis-Mediated Cell Adhesion in Nasopharyngeal Carcinoma

Luo Chen, Yiu Chun Chiang, Lai Sheung Chan, Wai Yin Chau, Maria Li Lung, Michael Kahn, Kwok Wai Lo, Nai Ki Mak and Hong Lok Lung

Supplementary Methods and Figures

1. Supplementary Materials and Methods

1.1. Wound Healing Assay

Wound healing assay was conducted as previously described [67]. C666-1 cells were allowed to grown to full confluency and the cell monolayer was scratched with a pipette tip to generate wounds. ICG-001 or corresponding solvent were added and the cells were incubated for 36 h. Mitomycin C (10 µg/mL) was used in both control and treatment groups to block the cell proliferation [68]. Images of wounds were captured at time 0 h, 18 h, and 36 h. Wound area were then analyzed with Image J. The relative recovery was calculated as:

(wound area after incubation / wound area at time 0 h) × 100

1.2. Screening of EMT and CSCs Related miRNAs

Total RNA from each treatment group was extracted using TRIZOL® reagent (Invitrogen). qRT-PCR using SuperScript™III Reverse Transcriptase (Invitrogen) was performed for the detection of 5S rRNA for data normalization. For detection of miRNAs, reactions were carried out with TaqMan MicroRNA Assays (Life Technologies/Thermo Fisher Scientific, MA, USA) according to the manufacturer's instructions. The assays employed pre-designed, target-specific stem-loop reverse transcription miRNA primers (Thermo Fisher Scientific) for the mature miRNAs. All qRT-PCRs were performed in triplicates on an ABI 7500 real-time PCR system (Applied Biosystems, Foster City, CA, USA) as instructed by the manufacturer.

1.3. CSCs-enriched Tumor Spheres Formation Assay

CSCs-enriched tumor spheres formation assay was used to assess the effects of miR-134 on the growth of CSCs-like cancer cells in NPC [69]. miR-134 transient transfection was conducted as described in Materials and Methods in the main text. miR-134 transfected or control NPC cells (1×10^4 cells/well) were seeded in DMEM/F12 (GIBCO) supplemented with EGF (20 ng/mL) (Sigma-Aldrich; Merck KGaA), FGF (20 ng/mL) (Cell Signaling Technology, Inc.), and IGF (20 ng/mL) (Cell Signaling Technology, Inc.) in 6-well ultra-low attachment plate (Corning). The cultures were then incubated in a humidified chamber at 37 °C for 7 days. The cultures were fed with fresh growth factors every 2 to 3 days. The images of all tumor spheres were captured under an inverted microscope and those with a diameter greater than 20 µm were analyzed by ImageJ software. Total numbers of tumor spheres formed per well from 2 – 3 independent experiments were analyzed with Student's *t*-test.

1.4. RNA Sequencing and Analysis

Transient overexpression of pre-miR-134 or pre-miR-Ctrl was conducted as described in the Materials and Methods part in the main text. Forty eight h after transfection, total RNA was isolated using TRIzol Reagent. Messenger RNA was purified from total RNA using poly-T oligo-attached magnetic beads. The first strand cDNA was synthesized using random hexamer primers, followed by the second strand cDNA synthesis using dTTP for construction of cDNA libraries at Novogene. The libraries were checked with Qubit and real-time PCR for quantification and bioanalyzer for size distribution detection. The quantified libraries were then pooled and sequenced on an Illumina platform. The raw data (raw reads) of fastq format were firstly processed through in-house perl scripts (service provided by Novogene) to generate clean data (clean reads). All the downstream analyses were based on the clean data with high quality. Read mapping was performed with Hisat2 v2.0.5 and quantification of gene expression level was performed with featureCounts v1.5.0-p3. Expected number of Fragments Per Kilobase of transcript sequence per Millions mapped reads (FPKM) of each gene was then calculated based on the length of the gene and read count mapped to this gene. Differential expression analysis was performed with edgeR R package (3.22.5). The *p*-values were adjusted using the Benjamini & Hochberg method. A corrected *p*-value of 0.05 and absolute fold-change of 2 were set as the threshold for significantly differential expression. The clusterProfiler R package was then used for Gene Ontology (GO) enrichment and KEGG enrichment analyses. GO/KEGG terms with corrected *p*-values less than 0.05 were considered significantly enriched by differential expressed genes.

1.5. Immunohistochemical (IHC) Staining

IHC staining was performed as previously described [70]. The tissue sections were prepared with a thickness of 6 μm. Antigen retrieval was performed by boiling the slides in citrate buffer for 10 mins. Slides were then incubated with the integrin $\alpha 5\beta 1$ antibody (1:200 diluted) (Millipore, clone JBS5, #MAB1969) for overnight. Afterwards, the slides were incubated with a biotinylated goat anti-mouse secondary antibody for 30 mins. The slides were then incubated with streptavidin-peroxidase conjugate and subsequently reacted with 3,3'Diaminobenzidine (DAB) (Abcam, #ab64238) for detection.

2. Supplementary Figures

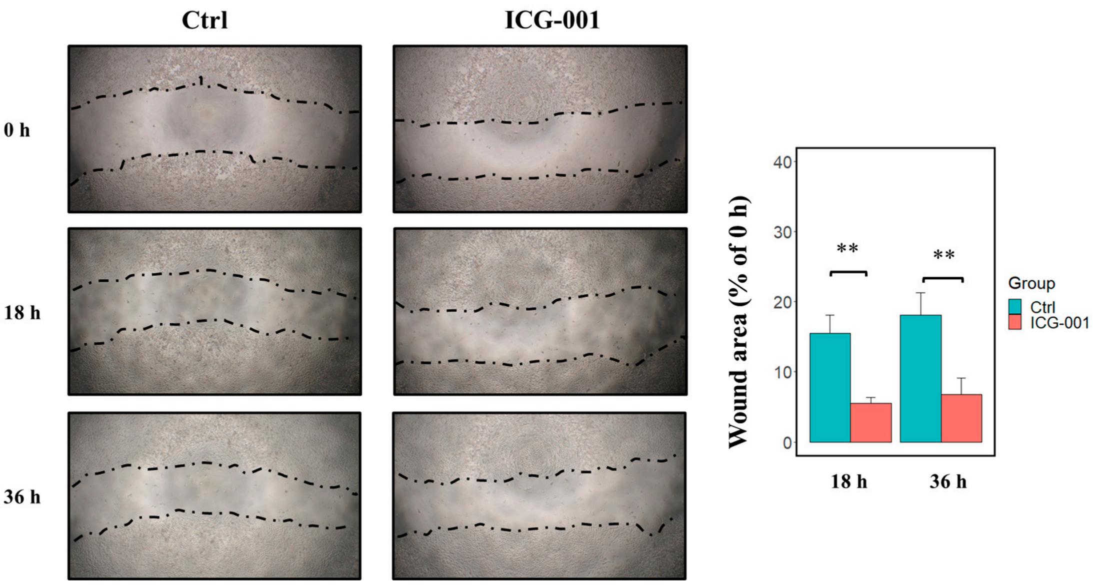

Figure S1. ICG-001 inhibits the wound healing of C666-1 cells. Representative images of the wounds are shown in the left panel. Recovery statistics from three independent experiments are summarized in the right panel. ** $p < 0.01$.

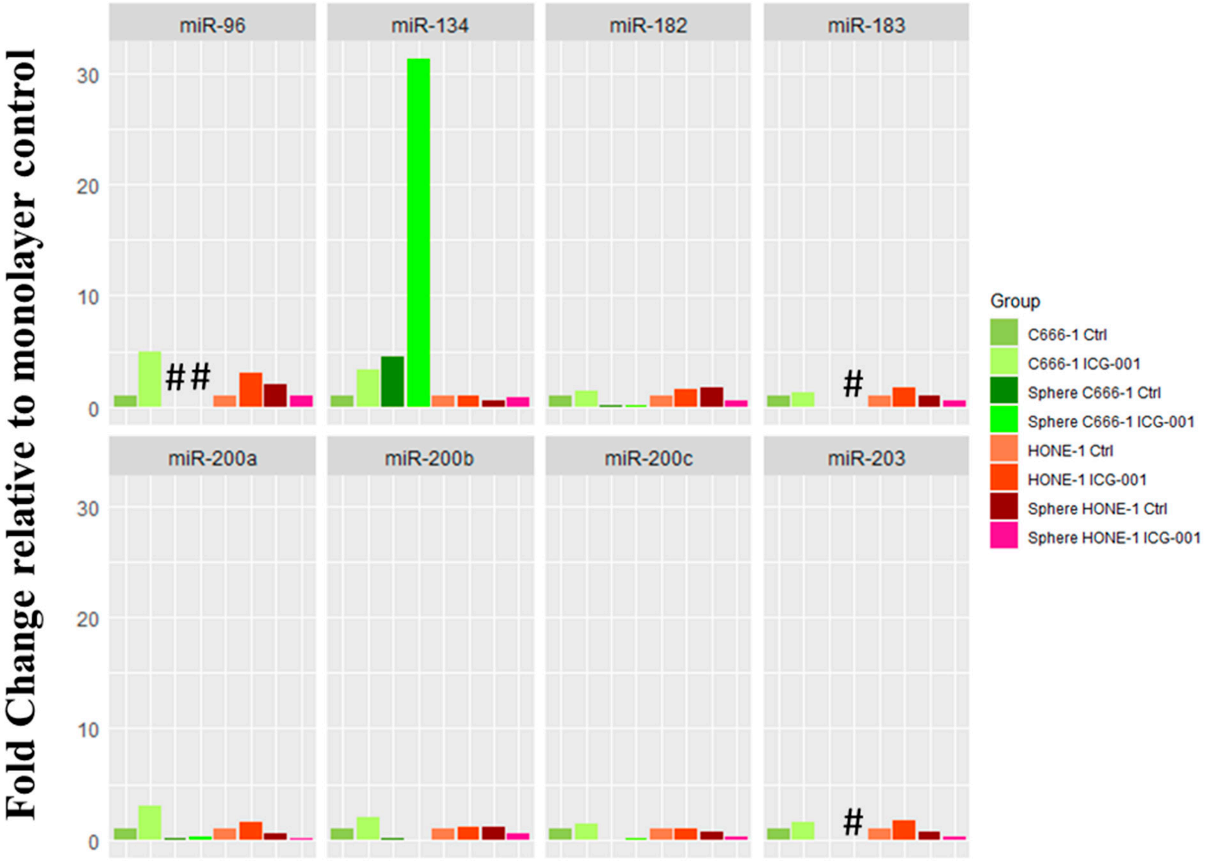

Figure S2. A screening on EMT and CSC related miRNAs identifies miR-134 as a target of ICG-001. C666-1 and HONE-1 from monolayer culture and CSC-enriched tumor sphere culture were treated with ICG-001 for one week. (#: expression was not detected).

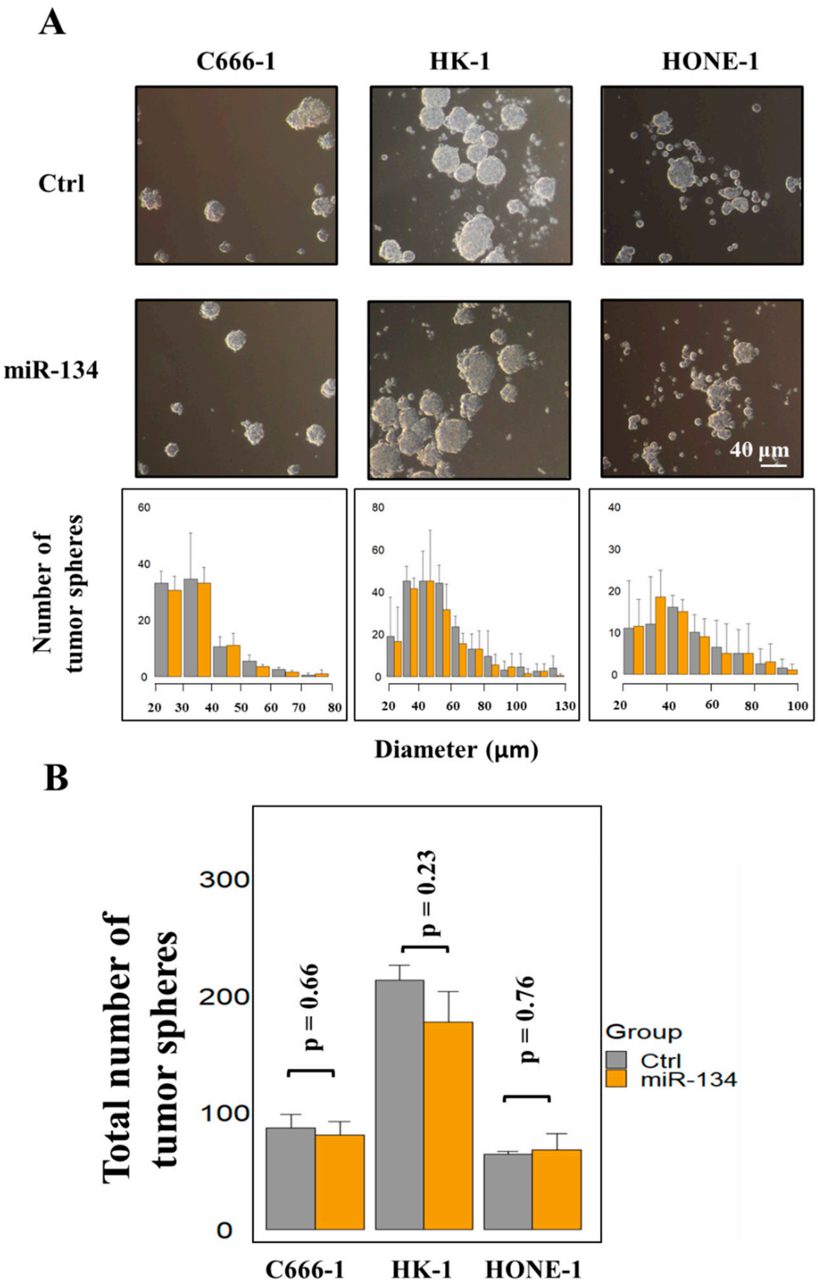

Figure S3. miR-134 does not affect the growth of CSCs-enriched NPC tumor spheres. (A) Representative photos of tumor spheres formed, with size distribution histogram at the bottom. **(B)** The total number of tumor spheres formed was not statistically different between control and miR-134 overexpressed NPC cells.

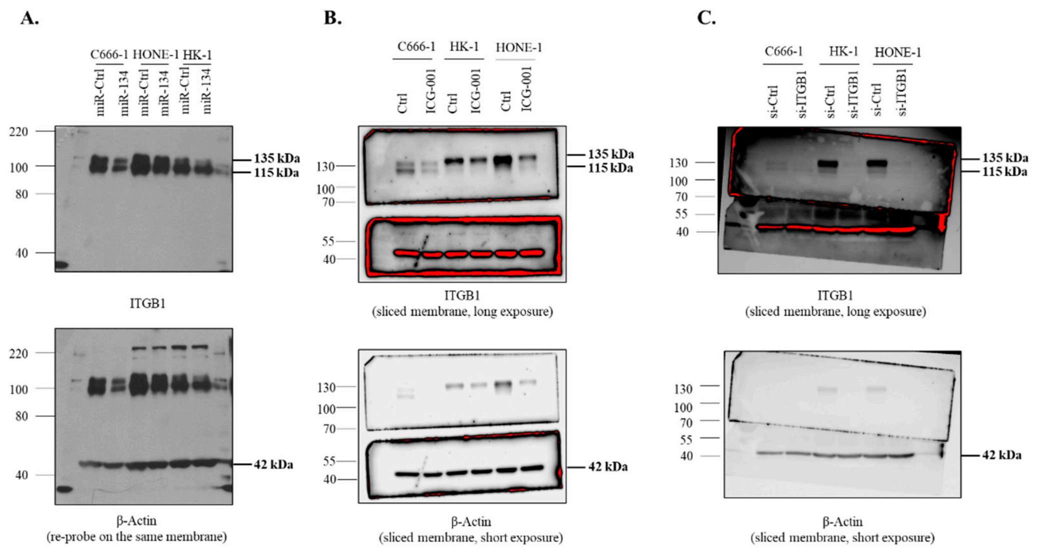

Figure S4. The whole blots of western blot data. **(A)** Overexpression of miR-134 in NPC cells reduced the expression of ITGB1. **(B)** ICG-001 treatment (10 μM, 3-day) inhibited the expression of ITGB1 in NPC cells. **(C)** Effects of si-ITGB1 on the expression of ITGB1 in NPC cells.

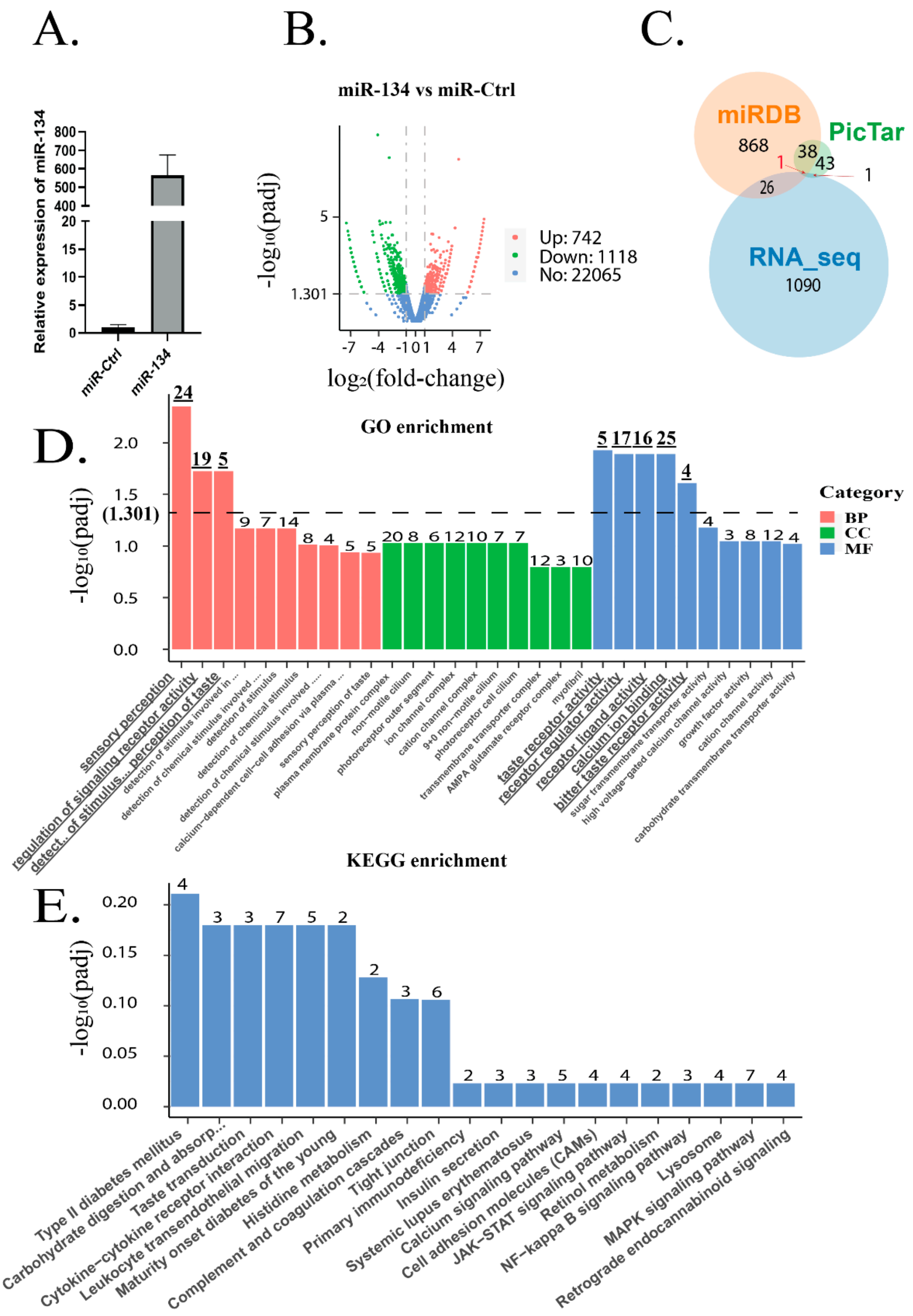

Figure S5. miR-134 is involved in modulation of receptors and taste sensory in NPC. **(A)** the efficiency of miR-134 transient overexpression in NPC C666-1 cells. Results were shown for three independent biological replicates. **(B)** RNA sequencing identified 1118 genes to be down-regulated and 742 genes to be up-regulated by miR-134 transient overexpression. Genes with corrected p-values < 0.05 and absolute $\log_2$(fold-change) values > 1 were considered as significantly different between miR-134 and miR-Ctrl. **(C)** Little overlap was observed between down-regulated genes detected via RNA sequencing and the predicted targets of miR-134 using PicTar and miRDB. **(D)** Gene ontology (GO) enrichment analysis for the down-regulated genes showed that the significantly affected biological processes occurred for the gene sets that are involved in 1) sensory perception, 2) regulation of signaling receptor activity, and 3) detection of chemical stimulus involved in sensory perception of taste. All the GO terms concerning cellular components were not significantly affected. The most

affected GO terms concerning molecular functions were related to taste activity, calcium ion binding and receptor ligand activity. The number of down-regulated genes concerning a particular GO term is labeled on top of each bar. BP, biological processes; CC, cellular components; MF, molecular functions. GO terms with corrected *p*-values less than 0.05 were considered significantly enriched by down-regulated genes. **(E)** Kyoto Encyclopedia of genes and genomes (KEGG) enrichment showed that all the KEGG metabolism-related terms were not significantly associated with down-regulated genes by miR-134 overexpression (corrected *p*-values less than 0.05 were considered significant). The number of down-regulated genes concerning a particular KEGG term is labeled on top of each bar. padj, adjusted *p*-value using Benjamini & Hochberg method.

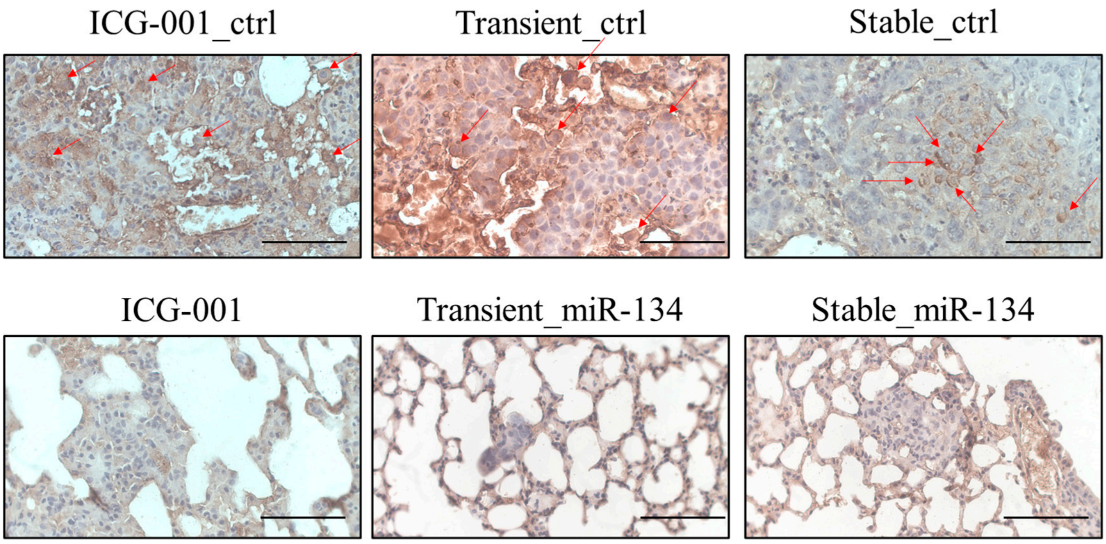

Figure S6. Expression of integrin α5β1 in lung-infiltrating NPC tumor cells. In the control groups, expression of integrin α5β1 was detected (red arrows); in the treatment groups, the tumor cells were negative for integrin α5β1 staining. Scale bar, 0.4 mm.
